# Recognition of freezing of gait in Parkinson’s disease based on combined wearable sensors

**DOI:** 10.1186/s12883-022-02732-z

**Published:** 2022-06-21

**Authors:** Kang Ren, Zhonglue Chen, Yun Ling, Jin Zhao

**Affiliations:** 1 System Informatics, Kobe University, Kobe, Hyogo Japan; 2 GYENNO SCIENCE CO., LTD., Shenzhen, Guangdong China; 3 Key Laboratory of Image Information Processing and Intelligent Control, Ministry of Education, and the School of Artificial Intelligence and Automation, Huazhong University of Science and Technology, Wuhan, Hubei China

**Keywords:** Parkinson’s disease, Freezing of gait, Sensor configuration, Feature selection

## Abstract

Freezing of gait is a common gait disorder among patients with advanced Parkinson’s disease and is associated with falls. This paper designed the relevant experimental procedures to obtain FoG signals from PD patients. Accelerometers, gyroscopes, and force sensing resistor sensors were placed on the lower body of patients. On this basis, the research on the optimal feature extraction method, sensor configuration, and feature quantity selection in the FoG detection process is carried out. Thirteen typical features consisting of time domain, frequency domain and statistical features were extracted from the sensor signals. Firstly, we used the analysis of variance (ANOVA) to select features through comparing the effectiveness of two feature selection methods. Secondly, we evaluated the detection effects with different combinations of sensors to get the best sensors configuration. Finally, we selected the optimal features to construct FoG recognition model based on random forest. After comprehensive consideration of factors such as detection performance, cost, and actual deployment requirements, the 35 features obtained from the left shank gyro and accelerometer, and 78.39% sensitivity, 91.66% specificity, 88.09% accuracy, 77.58% precision and 77.98% f-score were achieved. This objective FoG recognition method has high recognition accuracy, which will be helpful for early FoG symptoms screening and treatment.

## Introduction

Parkinson’s disease (PD) is a common neurodegenerative disease in middle-aged and elders. As of 2018, the global prevalence of PD was about 5.8 million, of which more than 50% patients were in China, and the number of new patients reached more than 100,000 each year [1]. Freezing of gait (FoG) is the most common and most disabling obstructive gait in the clinical manifestations of PD patients. FoG is mainly manifested in the transient block of motion: the patient is hesitant at the beginning, unable to walk or feels that his feet are stuck on the floor when walking, and is difficult to lift and step. The symptom usually lasts for several seconds, but occasionally can last as long as 30s. It often occurs when the patient starts walking, turns, and passes obstacles [2].

A survey of 6620 PD patients showed that 47% indicated that FoG often occurred, and 28% expressed that they experienced FoG every day [3]. FoG can easily lead to falls, fracture, disability and even death, which can seriously affect the quality of life of patients [4]. At present, the feasibility of FOG event detection has been investigated in many studies utilizing wearable sensors, such as force-sensitive shoe insoles [5], video cameras with 3D markers [6], and inertial sensors [7–11]. Among them, the inertial sensors have been intensively studied because of their low cost and compact size that enables easy wearing. Compared with vision-based methods, inertial sensors can monitor freely and are not restricted by tight spaces. In addition, studies have shown that the introduction of Rhythmic Auditory Stimulation (RAS) and visual cues can be used as auxiliary tools for FoG intervention [12, 13]. However, the drugs and surgical treatment are usually ineffective [14]. Therefore, early identification and evaluation of FoG is very important, and how to identify and screen FoG as early as possible in PD patient management is of great clinical significance. For the above reasons, it is necessary to develop wearable devices that can detect FoG.

### Related work

Recently, the machine learning algorithms are widely used to identify FoG. Mazilu et al. [9] deployed the online FoG detection and RAS intervention system to smartphones by using AdaBoost with C4.5algorithm on a public dataset collected from the 3D acceleration sensors attached to the shank, the thigh and to the lower back of 10 PD patients when they performed three varied walking tasks, which obtained more than 95% sensitivity and specificity with 10-fold cross-validation. Rodriguez-Martin et al. [8] used SVM to detect FoG through hand-crafted features calculated by a single waist-worn triaxial accelerometer from 21 PD patients when they perform the activities of daily living (ADLs), which achieved 88.1% sensitivity and 80.1% specificity through leave-one-subject-out (LOSO) validation.

In addition, deep learning algorithms have also been widely used in FoG detection. Ali Saad et al. [9] extracted the best features of different sensors fixed on the shin and the foot, and trained the Gaussian neural network from simulated dataset for classification to achieve an accuracy of 87% on independent test of 5 PD patients. Sigcha L et al. [10] used FFT transform of raw acceleration signals and combined long short-term memory (LSTM) to recognize FoG, which the signals were collected by a wearable inertial measurement unit (IMU) located at the waist when they did scripted ADLs. Their method achieved a significant improvement in the performance of FOG detection (leave-one-out validation: 87.1% sensitivity, 87.1% specificity) without increasing the length of the analysis window. Bohan Shil [11] gathered the data from three IMU sensors positioned at the ankles and spine of 67 PD patients when they performed TUG. They converted the recorded time-series sensor data into continuous wavelet transform scalograms and trained a Convolutional Neural Network to detect the freezing episodes, achieving an accuracy of 89.2% and a geometric mean (square root of sensitivity and specificity) of 88.8% on the independent test set.

A reliable FOG evaluation is still difficult, especially in daily life. More basic science and engineering research is therefore desired to improve the reliability of freezing evaluation, so multimodal FoG recognition system have been proposed. Luca Mesin et al. [15] found that the inertial sensors positioned on the lower limb are generally the most significant in recognizing FOG and they achieved 85% accuracy in the LOSO validation. Ying Wang et al. [16] used brain activity from EEG and motion data from accelerometers to detect FOG and found that the multimodal model performed better than the single-modal models. Another study [17] show that they obtained a sensitivity of 97%+/− 3%, a specificity of 96%+/− 7% through the LOSO validation when they used the multi-modal features.

Although the multimodal FoG recognition system may be effective, this system is complex to wear and is limited to experiments. Considering the cost and convenience of use, a trade-off must be made between installing the least number of sensors and collecting the most useful information. To this end, this paper designs related experiments to evaluate the FoG detection performance of different sensor configurations, compare the effectiveness of the two feature selection methods, develop the optimal combination of sensor configurations and select the optimal number of features in order to achieve the most effective system design.

## Materials and methods

### Materials

In materials, we introduce the dataset and sensors used in this study and describe the gait test.

### Dataset

In order to study the FoG of PD patients, the inclusion criteria are: (1) the study participants diagnosed with PD according to the Movement Disorder Society (MDS) diagnostic criteria (2) the participants determined with FoG according to clinical manifestation and FoG questionnaire; (3) the participants in the “ON” state of medication; (4) the participants that can walk for more than 20 m independently.

The exclusion criteria are: the participants had secondary PD causes, such as inflammatory, drug-induced, vascular, toxin-induced, etc., or participants with other neurodegenerative diseases, such as progressive supranuclear palsy or multiple system atrophy (MSA) and other Parkinson-plus syndromes.

This study recruited 12 Parkinson’s disease subjects who experienced FoG every day, including 10 males and 2 females. All subjects had a history of taking anti-PD drugs and had no cognitive impairment. The characteristics of the patients are presented in Table 1. This study was approved by the ethics committee of Ruijin Hospital, affiliated with Shanghai Jiaotong University School of Medicine.

**Table 1:** Characteristics of the included patients

	Value
**Number (female %)**	12 (16.7%)
**Age, year, (mean ± sd**^**a**^**)**	66.75 ± 4.95
**Age of Onset, years, (mean ± sd)**	26.90 ± 12.23
**Hoehn-Yahr Stage, N(%)**	(2.67 ± 0.51)
**2** **2.5** **3** **4**	2 (16.7%) 6 (50%) 3 (25%) 1 (8.3%)
**MDS-UPDRS**^**b**^**-III score, (mean ± sd)**	35.00 ± 10.02
**FOGQ**^**c**^ **score, (mean ± sd)**	13.17 ± 3.16

^a^*sd* standard deviation

^b^*MDS-UPDRS* Movement Disorders Society Unified Parkinson’s Disease Rating Scale

^c^*FOGQ* Freezing of Gait Questionnaire

### Test procedures

**Fig. 1:**
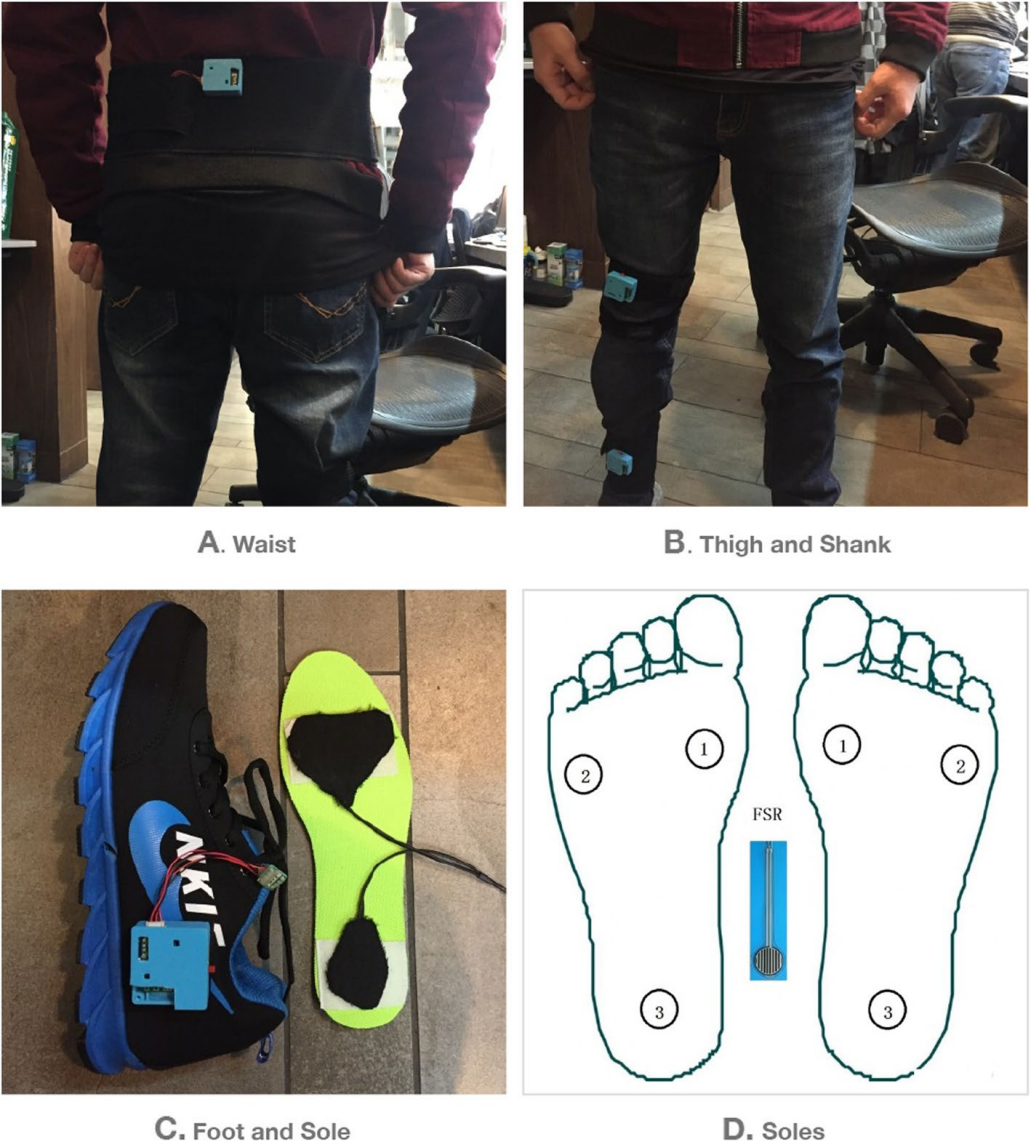
Sensors wearing location

Before the gait test, the inertial sensor (BMX055) was worn at the subject’s waist, thighs (above the knees), shanks (above the ankles), and feet (outer sides of the shoes). Six thin-film force sensor resistors (FSR) were integrated into the medial and lateral metatarsal region and heel region of the left and right insoles, respectively. The wearing locations of the above-mentioned sensors were shown in Fig. 1.

Subjects were asked to complete two specific gait tests to induce the occurrence of FoG episode: (1) random gait test; (2) Timed Up and Go test (TUG) [18]:the subject stood up from a armchair, walked straight forward to the turn line with yellow tape on it, turned 180 degrees and then walked back to the seat. The process was repeated more than 3 times for each subject. To avoid fatigue, subjects were allowed to take enough rest between tests.

The acceleration sensitivity is 4096 LSB/g (range -8 g ~ + 8 g). The sensitivity of the gyroscope is 16.4LSB/(dps) (range -2000dps ~ +2000dps). The data collected by the sensor is wirelessly transmitted to the PC software for collection and storage at a sampling frequency of 100 Hz. Bluetooth 4.0 protocol was adopted for wireless data transmission, and the maximum connection distance can reach 60 to 100 m. The researchers stood 3 m behind the subjects and recorded videos of the subjects’ movement. During synchronization, the PC software sent the timestamp to each sensor. After each sensor received the timestamp the PC software can synchronized the time with all sensors. When recording a video, we would ensure that the timestamp on the PC software was captured. In this way, time synchronization between video, PC and sensor can be achieved. After the test, the professional physicians watched the video records and marked the FoG episodes. The start of FoG episode was defined when the patient was instructed to initiate walking but had not yet performed any effective step forward at any point in time. The end of an episode was defined as the time when an effective step had been performed with a relatively normal length and swing, and the step also had to be followed by continuous normal walk [19].

## Methods

**Fig. 2:**
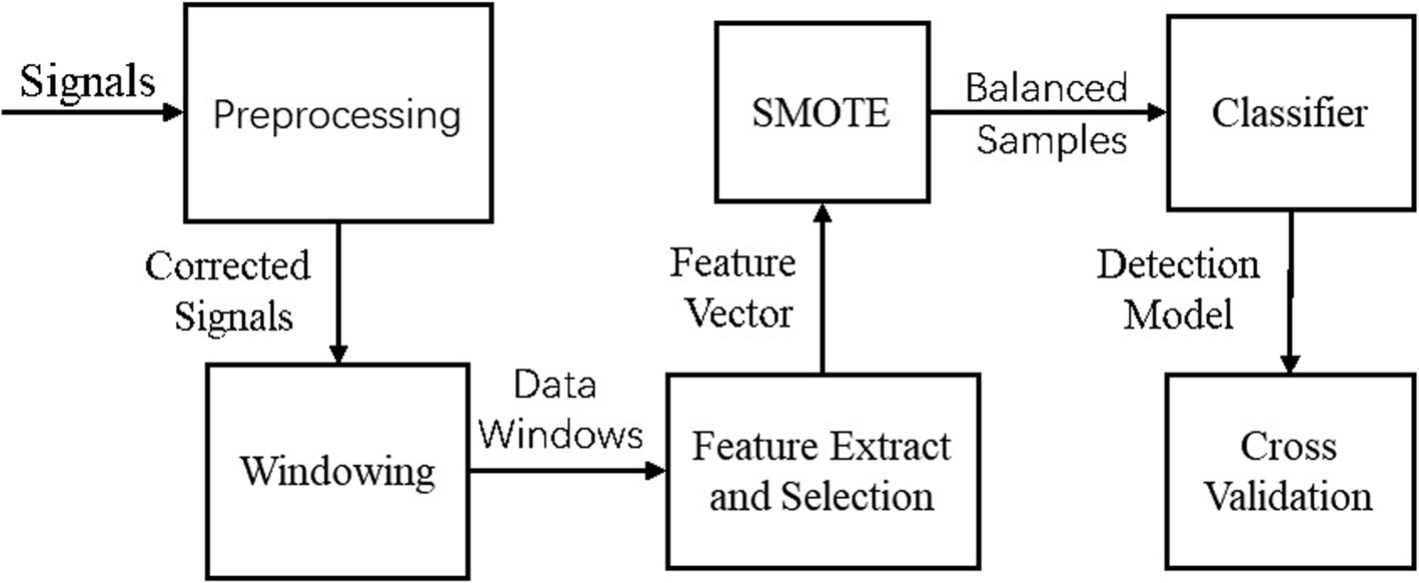
Modeling procedure

In methods, we present our modeling procedure for offline detection of FoG episodes (Fig. 2). After data pre-processing, the signals acquired by sensors were divided into several window data by windowing and then calculated features to obtain the sample set. Due to the suddenness of FoG, the sample is imbalanced. Therefore, in order to avoid the negative impact of imbalanced positive and negative sample ratios on the performance of the classifier, we performed random resampling method to rebalance the class distribution. Since Random under sampling deletes examples from the majority class and can result in losing information invaluable to a model for a small dataset, so we used Synthetic Minority Oversampling Technique (SMOTE) [20] to balance the training sample set. Finally, the balanced sample set was fed to Random Forest [21] Classifier to obtain the classification model. Mazilu et al. [22] compared several ML algorithms and reported that the best results were with the random forest algorithm; Rubén San-Segundo et al. [23] proved that the performance of random forest algorithm is close to that of the deep learning model. Therefore, we selected the random forest classifier in this study. We used LOSO cross-validation [24] method to evaluate the classifier, which divided the dataset into 12 folds according to the patients and the data of one patient will not appear in the training set and validation set in each fold, and the evaluation indicators are sensitivity, specificity, and accuracy.

### Data preprocessing

In the process of data collection, due to the impact of obstacles, the signal transmitted between the sensor and PC will be degraded and interrupted. When the connection was interrupted, we used the internal storage of the sensor to ensure the integrity of most data with only a small part temporarily missing. After collecting the sensor data, we performed data preprocessing. First, in order to fill in these missing values, linear interpolation was used. Then, the signal was filtered through a 26 order of equiripple FIR filter with the frequency range between 0.5 and 10 Hz to eliminate baseline drift.

### Windowing

The sensor data were divided into several window data by windowing for feature extraction. Specifically, when the sampling frequency is f (Hz), the window length is m seconds, and the step length is t seconds, after dividing the time window for l point collected by a certain sensor signal in a period, (l / f – m) / t + 1.

**Fig. 3:**
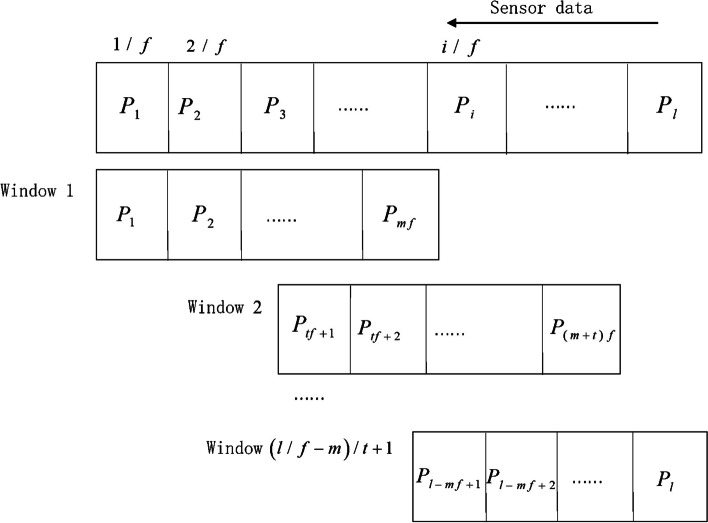
Windowing

Window data can be obtained. The windowing process is shown in Fig. 3. Previous studies showed that a window size of 2 s can yield a good result [25] and we think that 2 s is long enough to contain a step information. Therefore, the sliding time window is set to 2 s and the step length is set to 0.5 s. Since the observation time point of the subject’s FoG lags the actual FoG time point, so it is defined that the labeled result corresponding to the start time point of each window.

### Feature extraction

With the aim to obtain significant information from each window, some typical features (The description of each feature are shown in Table 2.) consist of time domain, frequency domain features and statistical features commonly used for FoG recognition were extracted from the sensor signal data. The above features can reflect the subjects’ movement information to some extent. For example, Moore et al. [26] proposed that the freezing index (FI) can detect FoG episodes according to changes in inertial signal power spectrum. And Mean value of each accelerometer axis measurements throughout the window gives the orientation of the inertial system related to gravity in absence of movement [8]. Standard deviation of each axis indicates the amount of movement performed in a window time.

**Table 2:** Features

Number	Feature	Description
**1**	**FI** ^**a**^	Ratio of [3, 8] Hz power (freezing zone) to [0.5,3] Hz power (motion zone)
**2**	**Energy**	The sum of squared amplitudes of signal after discrete Fourier transform
**3**	**Sum Power**	The sum power of the freezing zone and the motion zone
**4**	**Mean**	The mean value of the signal
**5**	**Absolute Mean**	Average of the absolute value of the signal
**6**	**Zero-crossing Rate**	The number of times the signal passes through the zero point
**7**	**Standard Deviation**	The mean square root of the sum of squares of deviation from mean
**8**	**Range**	The difference between the maximum and minimum value of the signal
**9**	**Root Mean Square**	The square root of the average of the squares of all values
**10**	**Maximum**	Maximum value of signal
**11**	**Minimum**	Minimum value of signal
**12**	**Principal Direction Eigenvalue**	Eigenvalue of covariance matrix
**13**	**Entropy**	Information uncertainty

^a^*FI* Freezing index

We used the signals of the inertial sensors (triaxial accelerometer and triaxial gyroscope) placed in 7 parts of the body and 6 signals of plantar pressure to calculate the above thirteen typical features. Therefore, (7*2*3*13 + 6*13) 624-dimensional feature vectors can be obtained. Since different units of each feature can affect subsequent feature importance evaluation, we use Min-max normalization method for data normalization. For every feature, the minimum value of that feature gets transformed into a 0, the maximum value gets transformed into a 1, and every other value gets transformed into a decimal between 0 and 1.

### Feature selection

The above high-dimensional feature space would reduce classification accuracy and high computational cost. In this paper, we used two filtering feature selection methods to select features on the training set – mutual information (MI) [27] and analysis of variance (ANOVA) [28].MI and ANOVA are typical filter feature selection methods that has low complexity, so it can quickly remove a large number of irrelevant features. Therefore, it is very suitable as a feature pre-filter. Yuqian Zhang [29] used these two feature selection methods to select features and then fed them to machine learning model, achieving an excellent FoG prediction result. Therefore, the importance of features was evaluated through these two feature selection methods and the effects of the two methods were compared as well.

In information theory, mutual information can measure the statistical dependence between two random variables, so it can be used to evaluate the relative utility of each feature for classification.

Theorem 1.

The Shannon entropy [30] of the discrete random variable W is defined as:

1 $$H(W)=-\sum \limits_Xp(w)\log p(w)$$

Then the mutual information [30] of the class label variable Y and the gait feature variable X can be expressed as:

2 $${\displaystyle \begin{array}{l}I\left(X,Y\right)\\ {}=H(Y)-H\left(Y|X\right)\\ {}=-\sum \limits_Xp(y)\log p(y)+\sum \limits_{y\in Y}\sum \limits_{x\in X}p\left(y|x\right)\log p\left(y|x\right)\end{array}}$$

In the above formula, H(Y) is a measure of the uncertainty about Y, and H(Y|X) is the uncertainty in Y when determining the observation quantity X. It can be seen that I (X, Y) is to reduce the uncertainty of the class label Y through the knowledge obtained at the gait feature X. Therefore, I (X, Y) can represent the amount of information of the class label Y contained in the gait feature X. A higher mutual information score indicates that the more classification information the feature contains, and the better the classification effect is.

In statistics, One-Way Analysis of Variance is a common method to study the relationship between sampling data and can be used to test the significant difference between class.

Theorem 2.

It is stipulated that represents a sample of the gait feature *X*_*k*_ ∈ {*X*_1_, *X*_2_, …, *X*_*M*_}, where *i* = 1, 2, …, *M* is the class number, and *j* = 1, 2, …, *N* represents the sample number of the i-th class. It is established in the null hypothesis that the class means of the gait feature *X*_*k*_ ∈ {*X*_1_, *X*_2_, …, *X*_*M*_} are equal, i.e., ${H}_0:{\mu}_1={\mu}_1=\dots {\mu}_M,{\mu}_i=\left(1/N\right){\sum}_{j=1}^N{x}_j^i$. The hypothetical result could be quantified by F test [31] that rejects *H*_0_ at *α* level as:

3 $$F=\frac{SS_b/\left(M-1\right)}{SS_w/\left({\sum}_{i=1}^MN-M\right)}>{F}_{\alpha}\left(M-1,{\sum}_{i=1}^MN-M\right)$$

In the above formula, *M* − 1 and ${\sum}_{i=1}^MN-M$ represent degrees of freedom, ${F}_{\alpha}\left(M-1,{\sum}_{i=1}^MN-M\right)$ is the (100*α*) th percentile of the F distribution. ${SS}_b={\sum}_{i=1}^MN{\left({\overline{x}}^i-\overline{x}\right)}^2$ represents the sum of variances between classes, ${SS}_w={\sum}_{i=1}^M{\sum}_{j=1}^N{\left({\overline{x}}^i-\overline{x}\right)}^2$ is the sum of variances within classes, ${\overline{x}}^i$ and $\overline{x}$ are the sample means of the classes and the population, respectively. F indicates the extent to which the sample of feature X is derived from the distribution of the same mean, and it can be determined whether there is a difference between the classes according to the feature values of different classes. The larger the F value, the greater the difference in the mean of features in different classes, thus providing useful information for feature selection.

### Sample balance

We used SMOTE algorithm [20] with 5 neighbors to increase the minority samples, so that the ratio of positive and negative samples after balance is 1.

The SMOTE algorithm is described as follows: for any one sample *x*_*i*_ in the class sample *X* = {*x*_1_, *x*_2_, *x*_3_, …, *x*_*i*_, …, *x*_*n*_}, its Euclidean distance from other samples is calculated, and k neighboring {*x*_*i*1_, *x*_*i*2_, *x*_*i*3_, …, *x*_*ij*_, …, *x*_*ik*_} of *x*_*i*_ are selected. According to the sampling rate m, m (*m* ≤ *k*)samples are randomly selected from {*x*_*i*1_, *x*_*i*2_, *x*_*i*3_, …, *x*_*ij*_, …, *x*_*ik*_}, and new data *x*_*ij*1_ is synthesized by linear interpolation for each sample,

4 $${x}_{ij1}={x}_i+\mathit{\operatorname{rand}}\left(0,1\right)\times \left({x}_{ij}-{x}_i\right)$$

In the above formula, *rand*(0, 1) is a random number between 0 and 1. Repeat the above process for each sample in X to synthesize data that is m times the original sample size.

### Modeling and verification

In this paper, the unified use of random forest algorithm was selected to classify FoG and non-FoG, and the results of different experimental designs were compared. We use grid search and LOSO methods to select the optimal estimator 10 to build a random forest model, and each tree was constructed with the maximum depth, so there was no need of pruning.

We evaluated the performance of the model using sensitivity, specificity, accuracy, precision and f-score. The following indicators were defined:

TP: Gait was correctly recognized as FoG;

TN: Gait was correctly recognized as non-FoG;

FP: Gait was incorrectly recognized as FoG;

FN: Gait was incorrectly recognized as non-FoG;

The calculation formulas for sensitivity, specificity, accuracy, precision and f-score are as follows:

Sensitivity (Recall): Sens. = TP/(TP + FN);

Specificity: Spec. = TN/(TN + FP);

Accuracy: Acc. = (TP + TN)/(TP + FN + FP + TN);

Precision: prec. = TP/(TP + FN);

F-score: 2*(Sensitivity *Precision)/(Sensitivity +Precision).

## Results

In order to get the most effective system design, we first selected the optimal feature selection method, then we chose the best sensor combination. Finally, according to the optimal feature selection method and sensor configuration, we feed the best features to the random forest model.

### Data research statistics

**Fig. 4:**
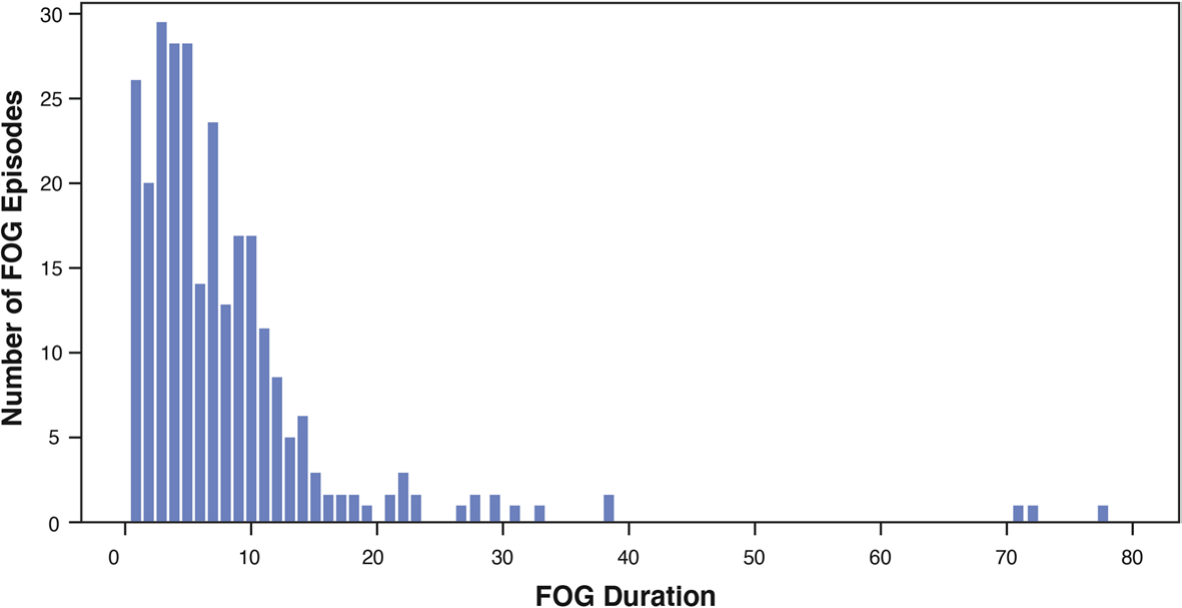
Distribution of FOG duration

This test recorded the data of 2 hours and 31 minutes in total. Ten out of 12 subjects showed FoG during the test, and 2 subjects behaved normally throughout the process. Professional physicians marked the total of 276 FoG episodes from the video record. The number of FoG episodes for each subject ranged between 0 and 27 (mean = 14.7, standard deviation = 9.3). The duration of FoG episodes ranged from 0.9 to 76.9 seconds (mean = 8.1 s, standard deviation = 9.3 s). More than 50% of FoG episodes lasted less than 6 seconds, whereas the majority (93.5%) lasted less than 20 seconds. The distribution of FoG durations was shown in Fig. 4.

### Feature selection method comparison

**Fig. 5:**
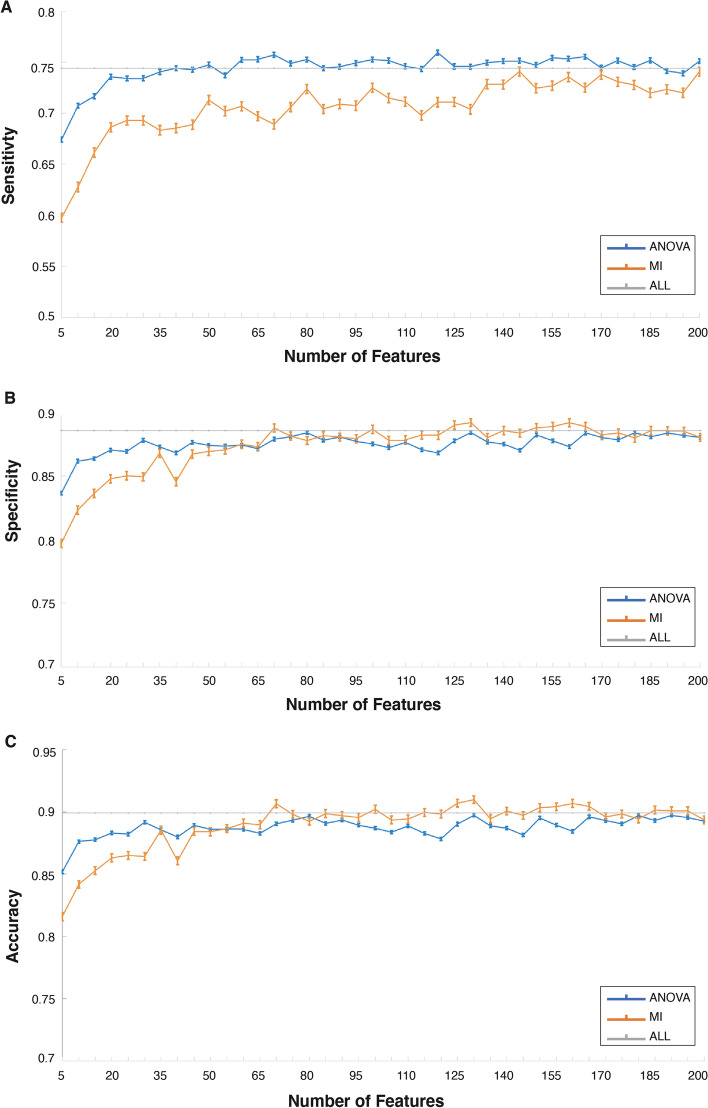
Comparison of the effectiveness between two feature selection methods (The vertical error bar denotes the standard deviation of cross-validation results)

Mutual information and ANOVA were used to calculate feature selection scores on 624-dimensional features, and the importance was ranked, followed by testing the classification effect of the features ranking top 5, top 10, top 15, ..., top 200 in importance under two methods respectively. The test results are shown in Fig. 5.

Figure 5 illustrates: The ANOVA feature selection method of the standard deviation of classification sensitivity and specificity is smaller than the mutual information method, which indicates higher stability. As can be seen from the three figures, the sensitivity, specificity, and accuracy of ANOVA feature selection method are all higher than that of MI feature selection method when only 5 features are used, and tend to be stable when fewer features are used, while the sensitivity, specificity and accuracy of MI feature selection method tend to be stable when more features are used, reflecting that the features selected by ANOVA method are more capable of classification. Considering the resource consumption of practical applications, we hoped to use as few features as possible, so a smaller number of features achieving better performance can meet our requirements. Therefore, ANOVA serves as a conducive feature selection method in this paper.

### Sensor configuration evaluation and feature selection

#### Sensor configuration evaluation

This paper first studied the optimal selection of a single sensor. In this regard, 13 features in Table 2 were extracted from the signals of each axis of 16 sensors for classification, and the detection results are shown in Table 3. According to the results, (1) left thigh accelerometer achieved optimal effect, and accelerometer and gyroscope on the shank and the waist also yielded positive detection results; (2) on the whole, sensors on the leg and the waist presented better detection effect than those on the foot and the sole, and the detection effect of accelerometer on the leg and the waist outperformed that of gyroscope. Since the thigh just above knee is the most inconvenient position to wear the sensor, so the shank (ankle) can be chosen as the actual position for placing the accelerometer or the gyroscope. (3) All precisions and f-scores ​​of a single sensor are low. The reason is that optimal performance may not be obtained with a single sensor, and imbalanced samples can lead to biased predictions.

**Table 3:** The effect of single sensor in detecting FOG

Sensor	Sensitivity (Recall)	Specificity	Accuracy	precision	F-score
**Left/right thigh accelerometer**	74.04% 71.90%	90.39% 89.27%	88.97% 87.76%	42.29% 38.95%	53.83% 50.53%
**Left/right shank accelerometer**	73.06% 71.58%	89.86% 89.15%	88.40% 87.62%	40.68% 38.62%	52.26% 50.17%
**Left/right thigh gyroscope**	72.08% 73.51%	89.88% 88.73%	88.33% 87.41%	40.45% 38.25%	51.82% 50.32%
**Left/right shank gyroscope**	71.99% 72.39%	88.95% 88.82%	87.47% 87.39%	38.38% 38.17%	50.07% 49.98%
**Waist accelerometer**	71.73% ——	88.85% ——	87.35% ——	38.19% ——	49.84% ——
**Left/right foot gyroscope**	71.26% 69.51%	88.21% 88.70%	86.73% 87.03%	36.64% 36.96%	48.40% 48.26%
**Waist gyroscope**	70.05% ——	88.46% ——	86.86% ——	36.62% ——	48.10% ——
**Left/right sole pressure sensor**	69.07% 68.83%	88.23% 87.96%	86.56% 86.29%	35.91% 35.35%	47.25% 46.71%
**Left/right foot accelerometer**	68.68% 68.15%	81.52% 88.24%	80.40% 86.49%	26.21% 35.61%	37.94% 46.78%

With regard to the optimal combination of multiple sensors, this paper took waist and left lower extremity as the research objects, and first evaluated the effect of sensor combination (accelerometer + gyroscope) at the same body part. Moreover, considering that FoG is a symptom mainly affecting the lower extremities of human body, the strategy of combining leg sensor + waist/foot/sole sensor was employed to probe into the combined effect of sensors at two parts. The detection results are shown in Table 4.

**Table 4:** FOG effect detected by multiple sensors

Sensor Position	Sensitivity (Recall)	Specificity	Accuracy	precision	F-score
**Left thigh**	76.02%	90.46%	89.20%	43.24%	55.13%
**Left shank**	76.73%	90.74%	89.52%	44.15%	56.05%
**Waist**	73.86%	91.89%	90.32%	46.49%	57.06%
**Left foot**	72.68%	89.58%	88.11%	39.92%	51.53%
**Waist + left thigh**	78.90%	91.32%	90.24%	46.40%	58.44%
**Waist + left shank**	78.17%	92.29%	91.06%	49.17%	60.37%
**Left thigh + left shank**	77.71%	90.88%	89.73%	44.91%	56.92%
**Left foot + left thigh**	77.12%	90.93%	89.73%	44.73%	56.62%
**Sole pressure + left thigh**	76.65%	90.04%	88.87%	42.42%	54.61%
**Sole pressure + left shank**	75.71%	90.48%	89.19%	43.22%	55.03%
**Left foot + left shank**	75.49%	91.40%	90.01%	45.66%	56.90%

According to the results, (1) in terms of sensor combination at the same part, the best result is obtained at the thigh and shank sensor; (2) in terms of sensor combination at different parts, sensor combination of waist + thigh and waist + shank demonstrate the best effect, revealing the potential of the waist sensor. (3) All precisions and f-scores of a part of low limbs and ipsilateral sensors are low. The reason is that optimal performance may not be obtained with a type of signal and ipsilateral sensors, and imbalanced samples can lead to biased predictions

#### Feature selection

**Fig. 6:**
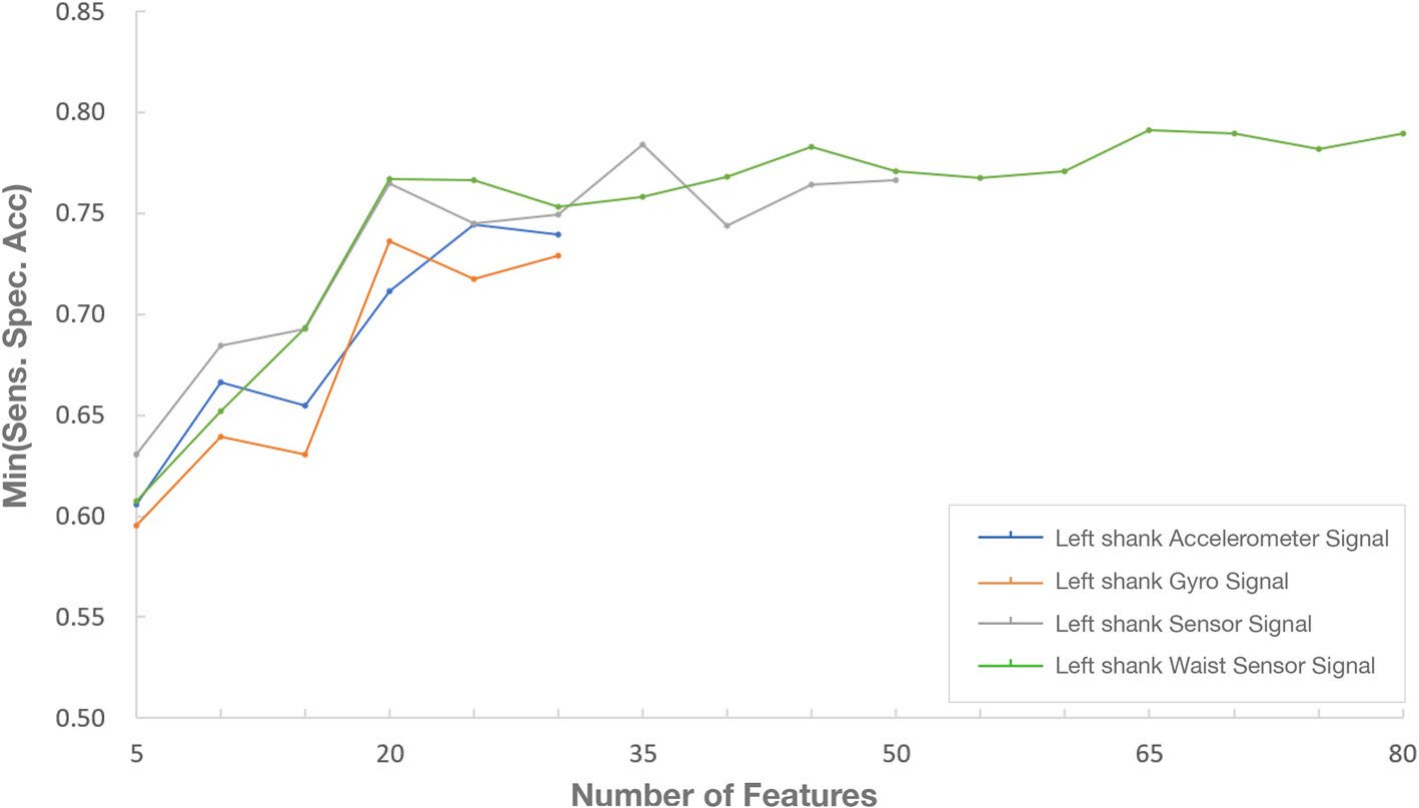
Classification effect of features selected for different sensor configurations

Considering information redundancy that may exist in the above sensor features, optimal features were selected for the left shank accelerometer, the left shank gyroscope, the left shank sensor combination, and waist & left shank sensor combination respectively by using ANOVA. Specifically, the importance ranking was performed first on each sensor features above, followed by testing the classification effect of top 5, top 10, top 15, ..., top k features in importance ranking. The test results are shown in Fig. 6.

According to the results, (1) under the same number of features, the combination of the left shank gyroscope and accelerometer can achieve better classification effect than a single sensor; (2) left shank accelerometer, left shank gyroscope, left shank sensor combination and waist + left shank sensor combination achieve the best classification results when using the features ranking top 25, top 20, top 35 and top 65 respectively, and the optimal classification effect achieved by left shank sensor combination is close to that achieved by sensor combination on waist + left shank. Taking into account overall factors including detection performance, cost and actual deployment requirements, left shank accelerometer and gyroscope were eventually chosen as the optimal sensor configuration. 35 features extracted are shown in Table 5, achieving 78.39% sensitivity (recall), 91.66% specificity, 88.09% accuracy, 77.58% precision and 77.98% f-score. In addition, according to Table 4, it is found that FI, entropy, root mean square, standard deviation, principal direction eigenvalue, and minimum value show good performance in most sensor axes. Therefore, when the number of sensor axes and the number of features is limited, these features can be prioritized.

**Table 5:** Features were calculated from left shank accelerometer and gyroscope

Accelerometer x axis	Accelerometer y axis	Accelerometer z axis	Gyroscope x axis	Gyroscope y axis	Gyroscope z axis
FI ^a^	FI	FI	Power	Max	FI
Variability	Min	Entropy	Energy	Range	Entropy
Standard Deviation	Mean		Variability	Root mean Square	
Entropy	Entropy		Entropy	Entropy	
Principal direction eigenvalue	Principal Direction Eigenvalue		Principal Direction Eigenvalue	Standard Deviation	
Absolute Mean			Absolute Mean		
Root Mean Square			Root Mean Square		
Energy			Standard Deviation		
Power			Min		
Range					
Max					
Min					

^a^*FI* Freezing index

## Discussion

Our study was conducted on 12 PD patients. Through experiments on feature selection methods, sensor configuration selection, and feature quantity selection, we obtained a set of optimal features that can be constructed a random forest FoG recognition model, finally achieving good recognition results: 78.39% sensitivity, 91.66% specificity, 88.09% accuracy, 77.58% precision and 77.98% f-score. The comparison of the results with most of the related literature work is difficult due to the diversity of approaches and validation methodologies.

Sigcha L et al. [10] identified the FoG of PD patients by deep learning method and got good results: 87.1% sensitivity, 87.1% specificity. Rodriguez-Martin et al. [8] detected FoG using support vector machines through a single waist-worn triaxial accelerometer with the result of 88.1% sensitivity and 80.1% specificity. Prithvi Patil et al. [32] used kinematic features calculated by accelerometer worn at 6 Lower limb joints to build an ELM-based multi-class gait classification model, achieving 93.54% overall classification accuracy. According to these evaluation results, our FoG recognition sensitivity does not significantly exceed their results, but it has a high recognition specificity. In addition, although previous studies compared the FoG recognition results of different algorithms, they did not attempt feature selection methods and sensor configurations, which may have a great impact on the performance of the model and the cost of the system.

Although a multi-sensor configuration (combining left shank sensor and waist sensor) has a better FoG recognition performance compared to a single-sensor configuration, considering recognition performance and actual deployment conditions, a single left shank sensor was chosen as the optimal sensor configuration. Luca Mesin et al. [15] found that the different lower limb sensor features have excellent performance in recognizing FOG. Ying Wang et al. [16, 17] also showed that the detection system using multi-sensor features (combining EEG and accelerometers) outperformed the single-sensor features (combining EEG and accelerometers). Though they also have good recognition performance, they didn’t consider the resource consumption problems.

At present, our study has some limitations. Our study only collected gait data of 12 PD patients in “ON” state, and the sample size was small, so we will increase research centers and continue to conduct FoG sampling including patients who are in “ON” state and further our study. Since this method only provides insight into the offline detection of FoG, and does not compare same other machine learning classifiers, therefore, the future research work will focus on online detection including timeliness of recognition, algorithm optimization and others. Also, due to the limited dataset, we did not verify the generalization performance of our algorithm on an independent test set, but we will further verify that our model is reliable in the near future.

## Conclusion

This paper studied the effect of different feature selection methods, discussed optimal sensor configuration for effective FoG recognition, and selected optimal features and quantity accordingly. Considering the actual deployment cost, computational cost, device portability and so forth, left shank gyroscope and accelerometer combination and 35 features were selected to detect FoG with 78.39% sensitivity, 91.66% specificity, 88.09% accuracy, 77.58% precision and 77.98% f-score. As the foundation for guiding subsequent implementation of measures, this research method can assist PD patients to resume walking and normal activities; it can also offer information about FoG symptoms as well, playing a guiding role in FoG research and treatment.

## Data Availability

All data as sociated with this study are available in the main text. Raw data cannot be publicly provided because of restrictions on the informed consent and risk of reidentification. More information of our algorithm can be obtained by submitting a request to research@gyenno.com.
